# Purulent Metritis

**Published:** 1892-01

**Authors:** O. J. Lanigan

**Affiliations:** Wenona, Ill.


### PURULENT METRITIS. *

By O. J. Lanigan.

The case which I have to report to you is one that I have encountered in my practice.

In April, '90, I was called to the country to deliver a mare of a foal. On my arrival at the farm, I found on inquiry that the owners of the mare and a farm-hand had been at work on the mare some four hours. They evidently wished to practice some themselves before calling any assistance. On examination I found the foal in the proper position, excepting left elbow, which was retaining it. On pressing the foal back and drawing on limb, it was an easy matter for her to foal, which she did with a little assistance, the foal being dead. On account of the rough usage she had received the vagina was very much swollen and painful, but otherwise the mare was in good condition. After using an antiseptic wash and giving the mare proper attention, I suggested to the owner that I should call the following day, as the mare would need medical care, but was informed that he could care for her himself.

I heard nothing of the mare until August, when I was told she was not doing well, and that she was discharging per vaginam.

In February, '91, the mare was brought to my hospital by her owner. He informed that after leaving her foal, she had always discharged more or less, and he had worked her during spring work ; but as she was getting worse he brought her to me. She presented a very poor appearance, terribly emaciated, dull eye, temperature 102 degrees F., pulse 55, did not care to eat. Abdomen enormously distended, and discharging, per vaginam, a dark colored offensive smelling fluid.

On examination per vaginam, about an inch anterior to meatus urinarius, I found the walls of vagina adherent, and that, anterior to adhesion, the vagina was filled with pus ; also that there was a sinus through the adhesion, although I was unable to find it.

---

* Case reported at the Annual Meeting of the Illinois Veterinary Medical Association, Nov. 18th, 1891.

I at once decided to try and break down the adhesion, after due preparation, with antiseptics. I took an eight-inch seton needle, and, passing right hand into the vagina, with left hand passed needle, directing its course with right, and as carefully as possible broke through the adhesion, which was about one-half inch in length. On withdrawing the needle, pus followed quickly. Forcing the index finger into the opening made with the needle, I enlarged the opening so that I passed the whole hand, when, on withdrawing my hand, an enormous quantity of pus followed. The mare began to strain violently, and with every strain forced pus from behind her—pus of a darkish cast and terribly offensive, driving my assistant and others out of the barn.

As soon as the mare stopped straining, I again passed my hand into vagina. The broken adhension left a jagged surface, which I dressed down with curved scissors. The os uteri and uterus I found more relaxed than any case I have seen immediately after foaling; the mare now began to show signs of pain, I at once prepared an antiseptic and thoroughly irrigated uterus, dressed the wound that I had made by placing a plegit of antiseptic cotton in the vagina, gave the mare a stimulant, clothed her warmly, and turned her in a box stall, where she at once lay down, and I then had hot rugs applied to her loins.

The next morning she could not rise, refused to eat, temperature 104 degrees F., pulse 60; gave stimulant, Tr. Mur. Iron, Quinia Sulph., with antiseptic washing of uterus and dressing to wound; also hot rugs continuously to loins; from second to sixth day no material change, only that she would occasionally eat a little with coaxing; nursed her as carefully as possible, and placed a careful man with her night and day.

On the sixth day she appeared much better, and with a little assistance got on her feet, but was very weak, and could with difficulty move her hind limbs. She now for the first time ate a mash and some hay, and appeared bright. After a thorough hand rubbing to limbs, she moved around the stall, though with difficulty. I now discontinued the use of all medicines excepting Fld. Ext. Hydrastis, and gave a soft diet. From this time on her recovery was rapid, and in one month from time of operation she left my hospital cured. She is now in fine condition and again in foal.

WENONA, ILL.

